# Vermeintlicher anaphylaktischer Schock während einer DJ-Anlage

**DOI:** 10.1007/s00101-025-01623-5

**Published:** 2026-01-16

**Authors:** Dorothea Heisig, Peter Spieth, Torsten Richter

**Affiliations:** Klinik f. Anästhesiologie & Intensivtherapie, Universitätsklinikum Dresden, Anstalt des öffentlichen Rechts des Freistaates Sachsen, Fetscherstraße 74, 01307 Dresden, Deutschland

## Anamnese

Bei einer 57-jährigen Patientin (170 cm, 90 kg) wurde ein *Cancer of Unknown Primary* (CUP) des Colon sigmoideum diagnostiziert. Vor der laparoskopischen Sigmaresektion wurde die Anlage eines Doppel J-Katheters (DJ) beidseits geplant.

Als Vorerkrankungen waren bekannt: arterieller Hypertonus, Asthma bronchiale, Depression, chronisches Schmerzsyndrom, Gräser- und Getreideallergie. Die bestehende Medikation umfasste Ramipril, Montelukast, Fluticason, Fenoterol bei Bedarf, Amisulprid, Bupropion, Hydromorphon und Pregabalin. Everolimus war seit einer Woche pausiert.

Vorherige Komplikationen bei einer Narkose zur Adnexektomie bzw. zur Entfernung von Peritonealkarzinoseherden wurden verneint.

## Befund

Klinisch imponierte ein gerötetes Gesicht. Die Patientin berichtete, durch das Tumorleiden emotional stark belastet zu sein. Sie wurde mit 1 mg Lorazepam per os (p.o.) prämediziert. Die Narkose wurde mit 15 µg Sufentanil intravenös (i.v.) und 160 mg Propofol i.v. eingeleitet und mit Sevofluran (minimale alveoläre Konzentration (MAC)0,8) inhalativ (inh.) geführt. Supportiv wurden 0,03 µg/kgKG und min Noradrenalin (NA) i.v. verabreicht. Eine Larynxmaske Gr.4 der 2. Generation konnte problemlos platziert werden. Die Patientin ließ sich problemlos manuell und druckkontrolliert beatmen (Spitzendruck p_PEAK_ 20 mbar, Inspiration:Exspiration I:E 1:1, Atemfrequenz AF 14/min, positiver endexspiratorischer Druck PEEP 3 mbar, inspiratorische Sauerstofffraktion F_I_O_2_ 0,5, Tidalvolumen V_T_ ≈ 300 ml, endtidaler Kohlenstoffdioxidgehalt etCO_2_ 5,3 kPa). Unmittelbar nach Gabe von 2 g Ceftriaxon i.v. wurde das eingestellte Drucklimit von 20 mbar erreicht, das V_T_ betrug weniger als 300 ml, auch bei erneuter manueller Beatmung. Der aufsichtsführende Facharzt wurde hinzugerufen und die Larynxmaske neu platziert. Anschließend imponierten ein weiterhin geringes V_T_ sowie eine starke Gesichtsrötung und -schwellung ohne Quaddelbildung oder Exanthem. Auskultatorisch zeigte sich ein ausgeprägtes exspiratorisches Giemen. Zudem war die Patientin hypoton mit Blutdruckwerten (RR) von 90/65 mm Hg. Die periphere Sauerstoffsättigung S_p_O_2_ fiel auf 91 % ab.

## Therapie und Verlauf

Die NA-Applikation wurde bis auf 0,15 µg/kgKG und min gesteigert und die F_I_O_2_ auf 1,0 erhöht. Daraufhin stieg die S_p_O_2_ auf 95 % an, die Hypotonie blieb bestehen. Bei Verdacht auf einen allergischen Schock auf Ceftriaxon erhielt die Patientin fraktioniert insgesamt 80 µg Adrenalin i.v., 100 mg Prednisolon i.v., 1 l kristalloide Infusionslösung i.v., 4 Hübe Salbutamol inh. und 0,5 mg Bricanyl subkutan (s.c.).

Die 10-minütige DJ-Einlage erfolgte parallel. Die Patientin wurde hyperton (200/110 mm Hg) und tachykard (125/min), aber Bronchospasmus, Gesichtsrötung und -schwellung waren nicht regredient. Zum Ende der Operation atmete die Patientin rasch spontan, erhielt jedoch bei anhaltender anaphylaktoider Symptomatik den zweiten Liter kristalloide Infusionslösung, 8 mg Dimetinden, 200 mg Cimetidin und 100 mg Theophyllin jeweils i.v. Eine Blutprobe zur Bestimmung der Mastzelltryptasewerte wurde abgenommen. Es erfolgte eine Inhalation mit 6 mg Adrenalin und 10 mg Salbutamol/250 µg Ipratropiumbromid. Zudem wurden erneut 100 mg Theophyllin i.v. verabreicht. Erst post inhalationem war der Bronchospasmus rückläufig, sodass die Patientin bei stabiler Spontanatmung extubiert und unter 12 l O_2_/min per Maske auf die anästhesiologische Intensivstation (ITS) verlegt wurde.

Während des ITS-Aufenthalts waren Bronchospasmus, Dyspnoe und Gesichtsschwellung rasch regredient. Sechs Stunden nach dem Initialereignis wurde die Mastzelltryptase im Serum erneut bestimmt.

## Diagnose

Nach der Narkoseeinleitung kam es zu einem signifikanten Anstieg der Beatmungsdrücke und einer ausgeprägten hypotensiven Entgleisung mit Verdacht auf einen allergischen Schock auf Ceftriaxon. Die erhaltenen Mastzelltryptasewerte im Serum waren jedoch normwertig. Somit ist die Anaphylaxie als Ursache der Episode eher unwahrscheinlich.

Ergänzenden anamnestischen Informationen zufolge handelte es sich bei dem genannten CUP um einen neuroendokrinen Tumor (veraltet: „Karzinoidtumor“) mit hepatischer Metastasierung. Die spezifischen Tumormarker (Chromogranin A, Serotonin im Serum (i. S.), 5‑Hydroxyindolessigsäure (5-HIES) im Urin (i. U.)) waren deutlich erhöht. Somit liegt die Vermutung einer Karzinoidkrise als Ursache der intraoperativen Symptomatik nahe.

## Diskussion

Neuroendokrine Tumoren (NET) sezernieren physiologisch aktive Substanzen wie u. a. Serotonin und Bradykinin. Erst bei hepatischer und pulmonaler Metastasierung oder extraintestinal gelegenem Primarius gelangen die Botenstoffe direkt in den Kreislauf und können so zum Karzinoidsyndrom (typische Trias: Diarrhö, Flush und Bronchospasmus) führen [2, 4, 7, 8]. Unter anderem bei emotionalem Stress, Narkoseeinleitung oder Manipulation am Tumor kann es zusätzlich zu extremen, vital bedrohlichen Blutdruckschwankungen kommen, die als Karzinoidkrise bezeichnet werden [11]. Ein ausführliches Studium der Krankenakte im Hinblick auf eine mögliche Metastasierung und die vorbestehende Symptomatik ist daher, auch vor kleinen operativen Eingriffen, unerlässlich.

Durch die sehr ähnliche klinische Präsentation ist die Unterscheidung zwischen einer Anaphylaxie und einer Karzinoidkrise schwierig. In beiden Fällen können Flush, Gesichtsschwellung, Bronchospasmus und hämodynamische Instabilität auftreten [4].

Im vorliegenden Fall wurde die Patientin zunächst unter der Annahme eines anaphylaktischen Schocks behandelt. Bei drohender Dekompensation von Atmung und Kreislauf wurde u. a. Adrenalin i.v. appliziert. Hier wäre primär eine intramuskuläre Injektion von 0,5 mg Adrenalin indiziert gewesen. Auch die Gabe von Theophyllin war laut aktueller Leitlinie obsolet [3].

Im Unterschied zur Anaphylaxie zeigen sich bei der Karzinoidkrise keine Hauteffloreszenzen wie Quaddeln oder Exanthem. Die Gabe von Katecholaminen kann die Symptomatik aggravieren ([1, 5]; Tab. 1). Auf Volumen‑, Salbutamol- und Katecholamingabe kann die Karzinoidkrise refraktär reagieren. Das fehlende Ansprechen auf die Anaphylaxietherapie bei Anamnese eines hepatisch metastasierten oder primär extraintestinal lokalisierten NET kann ein Hinweis auf das Vorliegen einer Karzinoidkrise sein [1, 2].

**Tab. 1:** Unterschiede zwischen Anaphylaxie und Karzinoidkrise^a^

	Anaphylaxie	Karzinoidkrise
Flush/Gesichtsschwellung	**+**	**+**
Quaddeln/Exanthem	**+**	–
Diarrhö	(+)	**+**
Bronchospasmus	**+**	**+**
Hämodynamische Instabilität	**+ **(RR ↓)	**+ **(v. a. RR ↓, RR ↑ mgl.)
Mastzelltryptase i. S.	**+**	–
Chromogranin A i. S./5-HIES i. U.	–	**+**
*Besserung durch* …		
Volumengabe	**+**	**(+) **Kann refraktär sein
Katecholamingabe	**+**	**(+) **Verschlechterung d. Symptomatik mgl.
β‑Sympathikomimetika inh.	**+**	**(+) **Verschlechterung d. Symptomatik mgl.
Anticholinergika inh.	**+**	**+**

^a^Übersicht über die auftretenden Symptome, Biomarker und Therapieoptionen bei Anaphylaxie vs. Karzinoidkrise. Die Einteilung erfolgt nach + (vorhanden), (+) (teilweise vorhanden) und – (nicht vorhanden).

Chromogranin A gilt als wichtigster Tumormarker der NET, unabhängig von deren Metastasierung. 5‑HIES hingegen ist häufig an der Entstehung einer Karzinoidkrise beteiligt [4]. Ex post wurde daher die Karzinoidkrise als Ursache der beschriebenen Episode vermutet. Eine Applikation von Katecholaminen hätte in diesem Fall eher vermieden werden sollen. Dennoch kann eine Anaphylaxie als Ursache der Symptomatik nicht mit Sicherheit ausgeschlossen werden.

Lange wurde eine massive Serotoninausschüttung als Ursache der Karzinoidkrise vermutet und daher eine Symptomkontrolle mit dem Somatostatinanalogon (SSA) Octreotid postuliert [1]. Bisher gibt es keinen Konsens zu einer prophylaktischen, perioperativen SSA-Gabe, lediglich verschiedene Empfehlungen [1, 2, 5–8, 11]:

- vor elektiven abdominellen Eingriffen: 3‑mal tgl. 100 µg Octreotid s.c. für Wochen,
- ab 12 h präoperativ: kontinuierliche Prophylaxe mit initial 50–100 µg/h Octreotid i.v., schrittweise Erhöhung bis zur ausreichenden Symptomkontrolle,
- Narkoseeinleitung: zusätzliche Bolusgabe von 50–100 µg Octreotid i.v.,
- bei intraoperativ auftretenden Symptomen: Bolusgabe von 50–1000 µg i.v.,
- SSA i.v. sollten für den Notfall jederzeit im OP zur Verfügung stehen.

In den letzten Jahren wird der Stellenwert der SSA bei der Behandlung der Karzinoidkrise, u. a. aufgrund des fehlenden Verständnisses der Pathophysiologie, zunehmend infrage gestellt und kontrovers diskutiert [6, 9, 10]. Weitere Studien zur genauen Pathophysiologie und Therapie der Karzinoidkrise sind dringend erforderlich.

## Fazit für die Praxis

- Karzinoidsyndrome und -krisen sind selten, aber potenziell lebensbedrohlich.
- Eine Abgrenzung zum anaphylaktischen Schock kann aufgrund der ähnlichen klinischen Präsentation schwierig sein.
- Das fehlende Ansprechen auf die Anaphylaxietherapie bei entsprechender Anamnese ist ein Hinweis auf ein Karzinoidsyndrom.
- Die präoperative Evaluation der Symptomatik und Metastasierung ist von großer Bedeutung.
- Bei sehr hohem Risiko für eine Karzinoidkrise kann perioperativ eine kontinuierliche i.v.-SSA-Gabe erforderlich sein. SSA sollten für den Notfall im OP zur Verfügung stehen.
